# The Voice of the Heart: Vowel-Like Sound in Pulmonary Artery Hypertension

**DOI:** 10.3390/diseases6020026

**Published:** 2018-04-13

**Authors:** Mohamed Elgendi, Prashant Bobhate, Shreepal Jain, Long Guo, Jennifer Rutledge, Yashu Coe, Roger Zemp, Dale Schuurmans, Ian Adatia

**Affiliations:** 1 Department of Obstetrics & Gynecology, University of British Columbia and BC Children’s & Women’s Hospital, Vancouver, BC V6H 3N1, Canada; 2 School of Electrical and Computer Engineering, University of British Columbia, Vancouver, BC V6T 1Z4, Canada; 3 Department of Pediatrics, Stollery Children’s Hospital, University of Alberta, Edmonton, AB T6G 2B7, Canada; prashantbobhate@gmail.com (P.B.); shreepal316@yahoo.co.in (S.J.); lguo1@ualberta.ca (L.G.); jcoe@ualberta.ca (Y.C.); 4 Division of Cardiology at Alberta Children’s Hospital, Calgary, AB T3B 6A8, Canada; Jennifer.Rutledge@albertahealthservices.ca; 5 Mazankowski Alberta Heart Institute, Edmonton, AB T6G 2B7, Canada; 6 School of Biomedical Engineering, University of Alberta, Edmonton, AB T6G 2V2, Canada; rzemp@ualberta.ca; 7 Department of Computing Science, University of Alberta, Edmonton, AB T6G 2E8, Canada; dale@cs.ualberta.ca

**Keywords:** congenital heart disease, hypertension, pulmonary, heart sounds, auscultation, machine learning, language recognition

## Abstract

Increased blood pressure in the pulmonary artery is referred to as pulmonary hypertension and often is linked to loud pulmonic valve closures. For the purpose of this paper, it was hypothesized that pulmonary circulation vibrations will create sounds similar to sounds created by vocal cords during speech and that subjects with pulmonary artery hypertension (PAH) could have unique sound signatures across four auscultatory sites. Using a digital stethoscope, heart sounds were recorded at the cardiac apex, 2^nd^ left intercostal space (2LICS), 2^nd^ right intercostal space (2RICS), and 4^th^ left intercostal space (4LICS) undergoing simultaneous cardiac catheterization. From the collected heart sounds, relative power of the frequency band, energy of the sinusoid formants, and entropy were extracted. PAH subjects were differentiated by applying the linear discriminant analysis with leave-one-out cross-validation. The entropy of the first sinusoid formant decreased significantly in subjects with a mean pulmonary artery pressure (mPAp) ≥ 25 mmHg versus subjects with a mPAp < 25 mmHg with a sensitivity of 84% and specificity of 88.57%, within a 10-s optimized window length for heart sounds recorded at the 2LICS. First sinusoid formant entropy reduction of heart sounds in PAH subjects suggests the existence of a vowel-like pattern. Pattern analysis revealed a unique sound signature, which could be used in non-invasive screening tools.

## 1. Introduction

When left untreated, pulmonary artery hypertension (PAH) is a progressively fatal disease [1]. Impacting an estimated 100 million people across the globe, PAH presents several complications that impact other diseases [2,3]. Because symptoms appear later in the course of PAH development, diagnosis is challenging, and often symptoms can be missed during the examination of patients.

Many investigators in the field of non-invasive PAH screening methodologies strive to detect the auscultation of an abnormally loud pulmonary component (P2) of the second heart sound (S2). These investigations have led to phonocardiographic exploration and the seeking of time-domain associations between the pulmonary artery pressure (PAp) and S2 [4,5,6,7,8,9,10,11]. With these investigations come the challenges of accurate demarcation, timing, and segmentation of the components of S2 [7,8,9,11,12,13]. 

Quantitative information on the frequency domain of heart sounds is explored in this paper, with the aim of differentiating between subjects with and without PAH [14]. The relative power of the frequencies between 21 Hz and 22 Hz of the heart sounds recorded at the 2^nd^ left intercostal space (2LICS), however, was not sufficient to capture PAH [14]. Recent results [15] showed that there is a reduced entropy of the first sinusoid formant of the heart sounds in children with PAH, suggesting the existence of an organized pattern in the 2^nd^ left intercostal space (2LICS) site. The analysis of this pattern revealed a unique sound signature, which could be applied to a non-invasive method to diagnose PAH. However, these results were obtained over a sample size of 27 subjects using only two sites: 2LICS and apex. This led us to further investigate two additional sites: 2^nd^ right intercostal space (2RICS) and 4^th^ left intercostal space (4LICS) to (1) determine if these two new sites also express the same reduction in entropy of the first sinusoid formant, (2) compare all four sites to identify the optimal site that is associated with reduced entropy indicating the existence of an organized pattern, and (3) examine the pattern existence over a larger sample size to validate our findings.

Normal speech patterns have a unique signature related to vocal cord vibration that can be used to make a distinction between certain traits (e.g., a male vs. female speaker). We postulated that unique sounds could be detected from the vibrations that originate in the movement of the pulmonary valve leaflets and pulmonary artery, in a manner similar to vocal cords. Through these sounds, a unique sound signature for PAH patients can be defined. 

Through formant (defined as the frequency resonance of sound and concentrations of energy that are prominent in a sound spectrogram) detection, we can ascertain the sound frequency spectrum [16]. First and second formant relative positioning can often be unique enough to differentiate between different speech sounds that reveal special qualities or timbres. Hence, the energy and entropy of the first formant of recorded heart sounds were investigated to determine if the heart sounds of subjects with and without PAH presented were significantly different.

We postulated that pulmonary circulation vibrations would create sounds similar to the sounds generated by the vocal cords during a speech, and that subjects with PAH will have a unique sound signature. 

## 2. Methods

The University of Alberta (UofA) Research Ethics Board approved the study. Informed and written consents were obtained from subjects (who were developmentally able) or guardians. 

**Figure 1:**
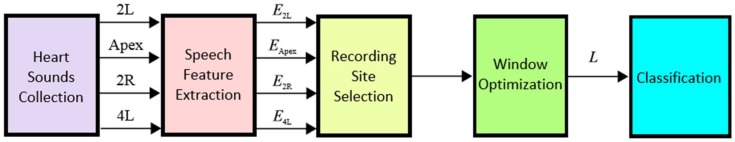
Flowchart of the speech-based pulmonary hypertension detection algorithm.

In this section, a new, speech-based, and numerically efficient algorithm is proposed to detect PAH in heart sounds based on the entropy of its formant. The structure of the proposed algorithm is shown in Figure 1. By developing a detector that depends on the vowel-like formant of the heart sound, it is predicted that the overall performance and detection accuracy will improve.

### 2.1. Heart Sounds Collection

Subjects undergoing right heart catheterization (required for managing an underlying cardiac condition) were approached. No subjects had an abnormal or prosthetic valve.

The PAp was measured directly and collected simultaneously with heart sounds which were obtained via fluid filled catheters. A 3M^TM^ Littmann^®^ 3200 electronic stethoscope (3M, Inc., Copenhagen, Denmark) was used to record heart sounds and Zargis Cardioscan^TM^ software (Zargis Medical Corp., Princeton, NJ, USA) was used for storing recorded heart sounds (*.wav mono audio format). Recorded heart sounds were obtained over 20 s with a sampling frequency of 4000 Hz. Heart sounds were recorded sequentially at the 2^nd^ left intercostal space (2LICS) and 2^nd^ right intercostal space (2RICS), 4^th^ left intercostal space (4LICS), and over the cardiac apical impulse. For signal analysis and optimization, MATLAB 2010b (The MathWorks, Inc., Natick, MA, USA) was used.

### 2.2. Definition of PAH

PAH, in adults and children, is defined as a mean pulmonary artery pressure (mPAp) ≥ 25 mmHg and a pulmonary artery wedge pressure (PAWp) or left atrial pressure (LAp) ≤ 15 mmHg measured at cardiac catheterization in subjects at rest [17,18,19]. The PAH classification follows the World Health Organization’s definition as published in [20,21].

### 2.3. Definition of Entropy

Entropy holds different meanings across different disciplines, therefore, we will define entropy in the context of this project. Entropy is defined as a measure of the disorder of the heart sound pattern. A lower entropy value suggests the existence of an organized heart sound pattern, while a higher entropy value indicates no existence of pattern. 

### 2.4. Heart Sounds Analysis

Classification for two groups was carried out based on whether or not each subject’s mean PAp was ≥ 25 mmHg or < 25 mmHg. All subjects had a PAWp < 15 mmHg. Heart sounds were analyzed by extracting the entropy of the first four sinusoids of the heart sound frequency bands. Two-sample *t*-tests were used to discover which recording site (the cardiac apex, 2R, 4L, or the 2LICS) was more informative for the diagnosis of PAH. 

### 2.5. Speech Feature Extraction

Extraction of unique heart sound signatures (defined as informants) was carried out using sine wave replicas. Through this process, sound patterns were distilled down to key elements through the removal of extraneous aspects. Heart sound recording tracks were transformed into sine wave replicas, as seen in speech analysis [22]. These sine wave heart sounds were then transformed by tracking the frequencies and amplitudes of the first four formants as they varied over time. A two-step process [15] was used to obtain the acoustic measurements. After resampling each sound file to 8 kHz, heart sound recordings were then broken into windows of 32 milliseconds each. An 8^th^ order linear-predictive-coding (LPC) analysis was applied to each window, which finds the coefficients of an 8^th^ order linear predictor (finite impulse response filter) that predicts the current value of the heart sound segment based on past samples. The four coefficients with the highest magnitudes were then converted to frequencies and magnitudes and stored in a data file. The resulting recordings each had an associated data file that included the eight parameters (four frequencies and four associated amplitudes) measured in each 32-millisecond window. This window captured information sufficient to track the change of the major formants in the original sound file over time. A synthesis routine [23] was then applied to data to produce four sinusoidal tones that varied over time. For each sinusoid (formant), the spectrogram (short-time Fourier transform) was calculated and referred to as *S* in the below equation. The entropy of a sinusoid as the power of the log-transformed spectrogram was calculated as follows:

(1) $$E_{f,k}=\sum_{i=1}^{L}\log(S_{f,i}^{2})$$

where *L* is the length of the processed heart sounds segment, *f* is the formant order: 1, 2, 3, or 4, *k* is the recording site: apex, 2RICS, 4LICS, or 2LICS, and *E* is the speech spectral feature for a specific recording site.

### 2.6. Recording Site Selection

After calculating the speech spectral feature (entropy of the sinusoid formant) for each of the four recording sites for 60 subjects, the speech spectral feature set contained 60 values: 35 values calculated from subjects with mPAp < 25 mmHg and 25 values from subjects with mPAp ≥ 25 mmHg. To demonstrate the significance of the mean of the samples within the speech spectral feature set, we compared the values by applying a two-sample *t*-test to compare the means, with *p*-value ≤ 0.05 considered significant. 

### 2.7. Window Length Optimization

Stationary heart sounds could theoretically be used as a whole to calculate the speech spectral feature (entropy of the first sinusoid formant), however, typically, these sounds are highly non-stationary and noisy. Given a heart sounds’ stationary nature, features can vary over time, thus making feature estimation from the entire 20-s signal no longer meaningful. A search was conducted over heart sound segment recordings to accommodate for potential non-stationarity recordings and to identify an appropriate window length, defined here as *L*. This allowed for accurate classification. Between 1 and 20 s window lengths were tested systematically. For each window length, the speech spectral feature was calculated and then averaged over all disjointed segments of length *L* within the duration of the 20-s heart sound recording. Note, the window length optimization was carried out on the most informative recording site as described in Figure 1.

### 2.8. Classification

For subject classification (PAH and non-PAH) an applied linear discriminant analysis (LDA) was used, based on the entropy of the first sinusoid formant of the heart sound of the most informative recording site. Classification performance was evaluated with LDA through leave-one-out (LOO) cross-validation, where each subject provided one case. Training sets were created by taking all cases except one, which was held out as a disjointed training set. Each training sets’ classification accuracy was determined by the single held-out test. The average accuracy over all *n* splits was then determined.

### 2.9. Statistical Tests

For each recording, the speech spectral feature (entropy of the first sinusoid formant) was calculated. Since there were 60 subjects, the speech spectral feature set contained 60 values: 35 values calculated from subjects with mPAp < 25 mmHg and 25 values from subjects with mPAp ≥ 25 mmHg.

The two-sample *t*-test was applied to establish the mean and median significance of the samples within the speech spectral feature set; here, the *p*-value ≤ 0.05 was considered significant. This procedure was repeated to determine the most informative window length for each feature over a range of window lengths of heart sounds (for the speech spectral feature, which carried from 1 s to 20 s; see the non-stationarity section above). 

Given that three different features were considered and that various window lengths settings were also considered simultaneously, it is plausible that a few *p*-values were small merely due to the stochastic fluctuations, rather than due to the systematic difference between subjects with mPAp < 25 mmHg and ≥ 25 mmHg. Consequently, the *p*-values needed to be appropriately corrected as implemented in [24,25]. To control the likelihood that a false positive may occur, the Bonferroni post-correction [26] can be used. Since multiple comparison tests (20 tests in total) were being dealt with, it was preferable to control for the false discovery (false positive) rate. Hence, the Holm-Bonferroni method was used since it controls the false positive rate while offering a simple test uniformly more powerful than the Bonferroni correction [27].

Two statistical measures were used for the output of the LDA analysis: sensitivity, calculated from the formula TP/(TP+FN), and specificity, calculated from the formula TN/(TN+FP), where TP is the number of true positives (PAH subjects detected as PAH subjects), FN is the number of false negatives (PAH subjects detected as normal PAp subjects), and FP is the number of false positives (normal PAp subjects detected as PAH subjects).

Finally, we conducted a post-hoc power analysis on the speech spectral feature calculated from the most discriminative recording site, to ensure that our sample size (60 subjects) was sufficient to draw meaningful conclusions. The power calculation was carried out using a two-sided *t*-test to have a power of 90% if the significance level was 0.05.

## 3. Results

Recordings were collected from 60 subjects (31 males and 29 females, median age of 7 years ranging from 3 months to 78 years of age). In Group 1, 35 subjects had a mean PAp < 25 mmHg (range 5–24 mmHg), and 25 subjects from Group 2 had a mean PAp ≥ 25 mmHg (range 25–93 mmHg). All subjects had a mean PAWp or left atrial pressure < 15 mmHg. No recordings or subjects were omitted from the analysis. Hemodynamic and clinical details of subjects are included in Table 1. The only statistically significant difference between the two groups were hemodynamic measurements reflecting the presence or absence of PAH. There was no difference in the PAWp or LAp or cardiac index between the two groups. The two groups did not differ statistically by age, weight, height, body surface area, or body mass index (Table 1).

### 3.1. Formant Extraction

**Figure 2:**
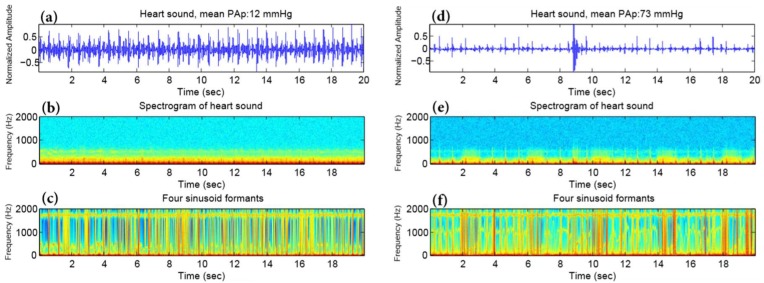
Spectrogram and four formants of heart sounds. (**a**,**d**) Heart sound recording, (**b**,**e**) spectrogram, (**c**,**f**) four sinusoid formants using the sine wave replica. At left, a subject with a mean pulmonary artery pressure of 12 mmHg, while at right a subject with a mean pulmonary artery pressure of 73 mmHg. In the bottom panel (four sinusoid formants), it is hard to distinguish the subject with pulmonary artery hypertension from the subject with normal pulmonary artery pressure. This contrasts with the bottom panel of Figure 6 in which the first sinusoid formant demonstrates a clear difference in the frequency distribution. Abbreviations: PAp = pulmonary artery pressure, sec = second, Hz = Hertz.

The middle panels of Figure 2 show an example of the spectrogram of the original heart sounds (Figure 2a,d) from a subject with a mean PAp < 25 mmHg (Figure 2b) and from a subject with a mean PAp ≥ 25 mmHg (Figure 2e) measured at the 2LICS. The Figure 2c,f shows the corresponding sine wave replica. The sine wave replica appears to have eliminated all extraneous information from the sound file other than the variation of the four formants over time.

The top panel of Figure 2 shows a spectrogram of heart sound, while the bottom panel shows a spectrogram of a synthetic sine wave replica of the heart sound. The left column represents a subject with mean PAp < 25 mmHg, while the right column represents subject with mean PAp ≥ 25 mmHg.

### 3.2. Formant, Recording Site, Window Length Selection

We investigated which out of the four sinusoid formants of heart sounds was more informative in all recording sites. As shown in Table 2, the first sinusoid formant was the most informative sinusoid for all heart sound recording sites, as it scored the *p*-value for distinguishing between subjects with and without PAH. Table 2 shows that the optimal site to discriminate between with and without PAH is the 2LICS as its *p*-value was the lowest (3.31 × 10^−9^) compared to those of the other three recording sites. 

**Figure 3:**
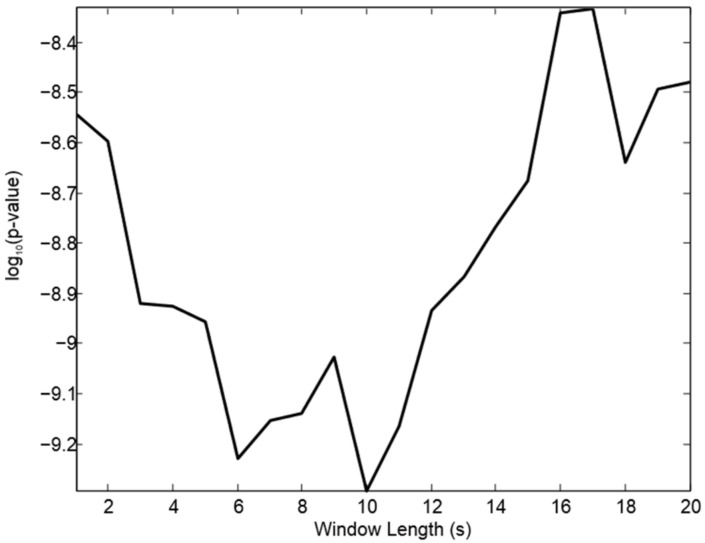
Influence of window length on the overall separability of subjects with pulmonary artery hypertension and without. A two-sample independent *t*-test was used, and reported *p*-values are corrected using Holm-Bonferroni method. We plotted the window length against the log to base 10 function of the corrected *p*-value to highlight the optimal length value.

We conducted a search over segments of the 2LICS heart sound recordings to identify the most appropriate window length for capturing a unique pattern (informative sinusoid formant). The significance, after statistical post-correction, was achieved after systematically testing a range of window lengths of the processed heart sound recordings (see window optimization section). As shown in Figure 3, the optimal window length for the entropy feature is 10 s, which significantly discriminates between subjects with and without PAH. It is worth noting that we plotted the window length against the log to base 10 function of the corrected *p*-value to emphasize the optimal length value.

**Figure 4:**
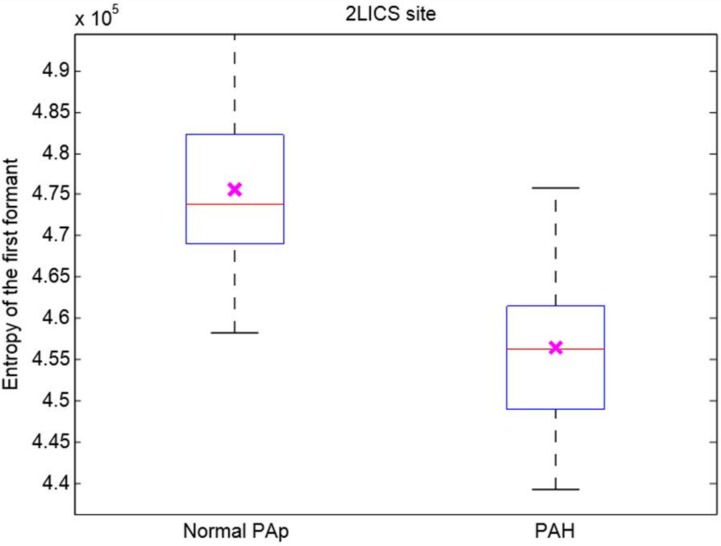
Boxplot of the entropy of the first sinusoid formant extracted from the heart sounds recorded at the 2^nd^ left intercostal space. The left box represents the entropy of the first sinusoid formant extracted from the heart sounds with a mean pulmonary artery pressure of 5–24 mmHg (*n* = 35), while the right box represents the entropy of the first sinusoid formant extracted from the heart sounds of children with a mean pulmonary artery pressure 25−93 mmHg (*n* = 25). The cross in each box refers to the statistical mean. Two-sample independent *t*-test was performed and a significant difference was detected between subjects with a normal pulmonary artery pressure and those with pulmonary artery hypertension (*p* < 0.05). Red line = median. Abbreviations: Normal PAp = normal pulmonary artery pressure, PAH = pulmonary artery hypertension.

The entropy of the first sinusoid formant with an optimal 10-s length of heart sounds recorded at the optimal 2LICS recording site of subjects with mean PAp < 25 mmHg was significantly lower than that of subjects with mean PAp ≥ 25 mmHg, as shown in Figure 4.

### 3.3. Linear Discriminant Analysis (LDA)

**Figure 5:**
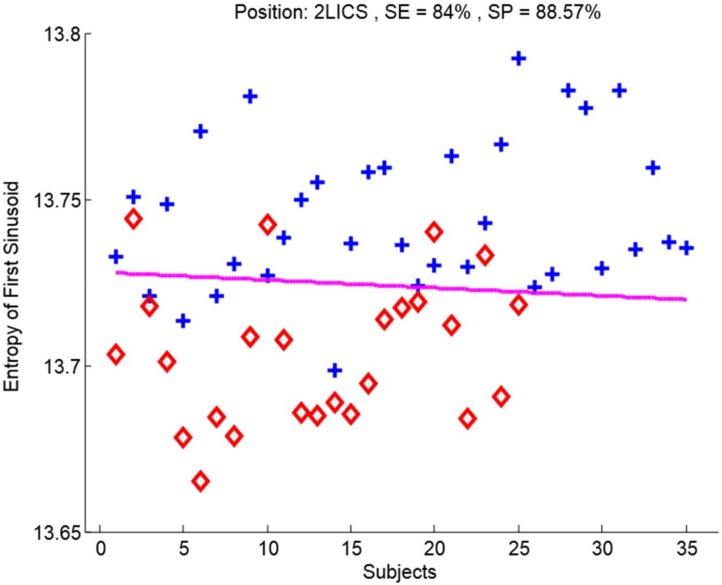
Classification of pulmonary artery hypertension. The entropy of the first sinusoid formant of heart sounds collected from the 2^nd^ left intercostal space site separated subjects with normal pulmonary artery pressure (blue crosses) from subjects with pulmonary artery hypertension (red diamonds). The pink line signifies the line of separation using linear discriminant analysis using the leave-one-out method. Abbreviations SE = Sensitivity, SP = Specificity.

We conducted LDA through LOO cross-validation on the recordings at the 2LICS with the optimal 10-s window. The entropy of the first sinusoid formant of the heart sounds incurred four false positive and four false negative results; see also Figure 5. The sensitivity of 84% and specificity of 88.57% of the entropy of the first sinusoid formant of the heart sounds to detect PAH were superior to those of the other three sinusoids. 

## 4. Discussion

In subjects with PAH (mean PAp ≥ 25 mmHg), entropy of the first sinusoid formant (within an optimized 10 s window length of the heart sound recordings at the 2LICS) was decreased significantly, with a sensitivity of 84% and specificity of 88.57%; see also Figure 3 and Figure 5. 

**Figure 6:**
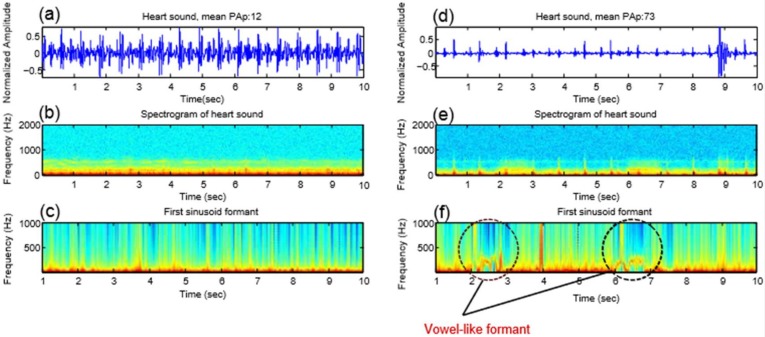
Spectrogram analysis and first formant of ten seconds duration of heart sounds. (**a**,**d**) Heart sound recording; (**b**,**e**) spectrogram; (**c**,**f**) first sinusoid formant using the sine wave replica. At left, a subject with a mean pulmonary artery pressure of 12 mmHg, while at right a subject with a mean pulmonary artery pressure of 73 mmHg. In the bottom panel, there is a clear vowel-like pattern in the frequency distribution representing low entropy in the subject with pulmonary artery hypertension. Abbreviations: PAp = pulmonary artery pressure, sec = second, Hz = Hertz.

The reduced entropy of heart sounds in subjects with PAH suggests the existence of an arranged pattern within the heart sounds. Using a non-invasive recording device, this pattern could be used to diagnose PAH using a non-invasive recording device. It can be seen in Table 2 that, generally speaking, first sinusoid formant entropy provided better separability between PAH and normal PAp subjects, in addition to being more informative, when compared to the energy of the first sinusoid formant. Low entropy suggests a distinctly ordered pattern in heart sounds of PAH subjects in the time domain analysis, as shown in Figure 6f.

In clinical settings where time is at a premium for health care practitioners, a 20 s short recording time for the collection of diagnostic data is helpful and practical, especially in pediatric clinics. The cooperation of infants and children is unpredictable and lasts for a limited amount of time, and quickly capturing much needed and valuable diagnostic information is an asset. All recordings collected from the 3M^TM^ Littmann^®^ 3200 digital stethoscope and 3M, Inc., Denmark electronic stethoscope, were included. This suggests that this analysis of the heart sounds in the frequency domain is robust. Higher fidelity sensors would likely improve the sensitivity and specificity value of the submitted results. 

Our signal analysis did not focus on timing, detection, or the splitting of S2 intervals between aortic and pulmonary components. There are significant challenges with the traditional PAH clinical indicators, such as seen with the differentiation between the aortic and pulmonary components of the S2 and the splitting interval [6,7,8,9,12,13]. Thus, in the paper, the main focus was on utilizing frequency-domain hidden voice patterns as opposed to typical time-domain markers [10] and relative power calculations of the narrow frequency band 21–22 Hz [14]. Heart sounds of PAH and non-PAH subjects were characterized, in a similar manner to the analysis and characterization of speech patterns, for the detection of unique vowel-like signature sounds generated by the heart with an increased PAp. Since registering the timing of heart sound recordings with right heart or pulmonary artery events is not needed, the proposed method is advantageous and simplifies current PAH non-invasive diagnostic approaches.

It is interesting to note that the recording site that best distinguishes patients with a mean PAp ≥ 25 mmHg from subjects with a mean PAp < 25 mmHg was the 2LICS, which is the traditional area for auscultation of pulmonary artery events.

## 5. Vowel-Like Visualization

In human spoken words, the vowel formants appear as “harmonic stacks” that mark the arrival of the glottal pulse train, and the spacing of the harmonics is clearly much wider in the high-pitched vs. the low-pitched vowels [16]. Figure 2c,f shows the four formants of the processed heart sounds; however, the harmonics are not exactly horizontally stacked to form a pattern. Interestingly, the first formant only provides an apparent pattern, as shown in Figure 6f, in the case of PAH. We noted that the pitch of these vowel-like sounds in Figure 6f was not perfectly steady. Regardless, it is quite clear that there is an organized pattern compared to the normal PAp case.

We found in the case of PAH that the vowel-like sound has a very prominent first formant harmonic around 300 Hz; however, there is little sound energy between 200 and 700 Hz. This phenomenon has been similarly found in the human /e/ and /i/ vowels [16]. Results were as excepted since our hypothesis was based on a previous observation where an extra heart sound was heard in PAH subjects using the digital stethoscope. This extra sound that we heard has been quantified mathematically and our algorithm was able to capture it and visualize it as shown in Figure 6.

Investigation of PAH was the main focus of this research, perhaps looking into different heart valve diseases using the same methodology would be interesting. We may be able to find different vowel-like patterns for each abnormality. Note, in our study here we did not have any subjects with abnormal or prosthetic valves.

## 6. Study Limitations

A larger sample size is needed to confirm the findings of this study. We studied 60 subjects. We conducted a post-hoc power analysis on the speech spectral feature with the smallest post corrected overall *p*-value, which is the entropy of the first sinusoid formant derived from the heart sounds (see Figure 3). The mean and standard deviation of the null hypothesis of the control group (subjects with normal PAp) are 4.75 × 10^5^ and 9.85 × 10^3^, respectively; while the mean under the alternative hypothesis (subjects with PAH) was 4.56 × 10^5^. The power calculation of the speech spectral feature from the 2LICS suggests that the representative sample size of the population should be ≥6 subjects for a power of 90% when the significance level is 0.05, which indicates that our sample size (60 subjects) was sufficient to draw meaningful conclusions. 

Prospective recordings with the investigators blinded to the patients’ diagnoses are also required in future studies. However, the use of LDA and LOO to analyze the findings removes investigator bias considerably. Since we had a small sample size, it was difficult to investigate whether the vowel-like pattern was more salient in adults or infants. However, this is one of the points that needs to be addressed in future research in this area.

## 7. Conclusions

The entropy (of the first sinusoid formant) of heart sounds in PAH subjects, recorded via a handheld digital stethoscope and recorded simultaneously with direct pulmonary artery pressure measurements, was significant and revealed a mean PAp ≥ 25 mmHg, yielding a classification sensitivity of 84% and specificity of 88.57%. An optimized window length of 10 s, within a 20-s recording of the heart sounds at the 2LICS, was applied. Results suggest that heart sounds contain organized vowel-like patterns similar to the organized patterns found in speech analysis. This pattern is a unique sound signature produced by the hypertensive pulmonary artery and right ventricle. 

More work is needed to generalize the findings, such as conducting similar research on a larger sample size, allowing for a more informed collective decision based on multiple heart sounds. We suggest that the entropy of the first formant be used as a screening tool, as results show potential for providing insight into screening for PAH cases, when compared to healthy subjects. Another point for consideration is trying different digital stethoscopes to confirm the scalability of the findings over different devices. 

## Figures and Tables

**Table 1:** Comparison of clinical and hemodynamic data between subjects with pulmonary artery hypertension (mean PAp ≥ 25 mmHg) and subjects with normal pulmonary artery pressures (mean PAp < 25 mmHg).

	Normal Pap Mean (std)	PAH Mean (std)	*p*-Value (*t*-test)
Age	14.1 (18.0)	11.9 (17.4)	0.64
Height	120.9 (42.0)	120.0 (36.5)	0.93
Weight	35.7 (29.1)	32.8 (25.5)	0.69
Body Surface Area	1.1 (0.6)	1.0 (0.5)	0.77
Systolic Pulmonary Artery Pressure	24.6 (5.6)	69.2 (26.8)	1.42 × 10^−13^
Diastolic Pulmonary Artery Pressure	9.3 (2.9)	30.2 (15.3)	9.96 × 10^−11^
Mean Pulmonary Artery Pressure	15.3 (4.1)	47.4 (19.3)	1.31 × 10^−13^
Pulmonary Vascular Resistance Index	2.0 (1.0)	12.1 (6.2)	2.96 × 10^−13^
Pulmonary Blood Flow Index	4.4 (2.5)	3.4 (1.0)	0.06
Mean Left Atrial Pressure	7.3 (3.4)	8.8 (5.0)	0.19
Mean Right Atrial Pressure	4.2 (3.1)	4.4 (2.2)	0.77
Systolic Blood Pressure	92.5 (20.4)	93.2 (22.7)	0.89
Diastolic Blood Pressure	53.0 (17.5)	54.2 (15.3)	0.78
Mean Blood Pressure	69.0 (18.3)	70.2 (17.2)	0.81
Heart Rate	95.4 (23.3)	87.6 (24.3)	0.21
Electrocardiogram QRS Duration in Lead V1	99.7 (22.9)	92.4 (21.3)	0.21
Electrocardiogram PR Interval in Lead 2	124.7 (37.7)	119.3 (36.6)	0.58

Abbreviations: Normal PAp = normal pulmonary artery pressure, PAH = pulmonary artery hypertension.

**Table 2:** Formant Selection. A two-sample independent *t*-test was performed and a significant difference was detected between subjects with a normal pulmonary artery pressure and those with pulmonary artery hypertension. It is clear that the first formant is more informative than other three formants as it scored the lowest *p*-value among all of them. Moreover, the 2LICS is more informative compared to the other three recording sites. Abbreviations: 2LICS = 2^nd^ left intercostal space, 2RICS = 2^nd^ right intercostal space, and 4LICS = 4^th^ left intercostal space.

	2LICS	Apex	2RICS	4LICS
Formant	*p*-Value	*p*-Value	*p*-Value	*p*-Value
1	3.31 × 10^−9^	7.38 × 10^−2^	2.81 × 10^−3^	1.03 × 10^−1^
2	0.12	0.06	0.19	0.56
3	0.02	0.19	0.61	0.14
4	0.05	0.89	0.83	0.79

## References

[1] Humbert M., Sitbon O., Chaouat A., Bertocchi M., Habib G., Gressin V., Yaïci A., Weitzenblum E., Cordier J.-F., Chabot F. (2010). Survival in patients with idiopathic, familial, and anorexigen-associated pulmonary arterial hypertension in the modern management era. Circulation.

[2] Butrous G., Ghofrani H.A., Grimminger F. (2008). Pulmonary Vascular Disease in the Developing World. Circulation.

[3] Adatia I., Kothari S.S., Feinstein J.A. (2010). Pulmonary hypertension associated with congenital heart disease: Pulmonary vascular disease: The global perspective. Chest.

[4] Leatham A. (1957). Splitting of heart sounds and a classification of systolic murmurs. Circulation.

[5] Leatham A. (1954). Splitting of the first and second heart sounds. Lancet.

[6] Longhini C., Baracca E., Brunazzi C., Vaccari M., Longhini L., Barbaresi F. (1991). A new noninvasive method for estimation of pulmonary arterial pressure in mitral stenosis. Am. J. Cardiol..

[7] Chen D., Pibarot P., Honos G., Durand L.-G. (1996). Estimation of pulmonary artery pressure by spectral analysis of the second heart sound. Am. J. Cardiol..

[8] Xu J., Durand L.-G., Pibarot P. (2002). A new, simple, and accurate method for non-invasive estimation of pulmonary arterial pressure. Heart.

[9] Nigam V., Priemer R. (2006). A dynamic method to estimate the time split between the A2 and P2 components of the S2 heart sound. Physiol. Meas..

[10] Elgendi M., Bobhate P., Jain S., Rutledge J., Coe J.Y., Zemp R., Schuurmans D., Adatia I. (2014). Time-domain analysis of heart sound intensity in children with and without pulmonary artery hypertension: A pilot study using a digital stethoscope. Pulm. Circ..

[11] Chan W., Woldeyohannes M., Colman R., Arand P., Michaels A.D., Parker J.D., Granton J.T., Mak S. (2013). Haemodynamic and structural correlates of the first and second heart sounds in pulmonary arterial hypertension: An acoustic cardiography cohort study. BMJ Open.

[12] Tranulis C., Durand L.-G., Senhadji L., Pibarot P. (2002). Estimation of pulmonary arterial pressure by a neural network analysis using features based on time-frequency representations of the second heart sound. Med. Biol. Engin. Comput..

[13] Dennis A., Michaels A.D., Arand P., Ventura D. (2010). Noninvasive diagnosis of pulmonary hypertension using heart sound analysis. Comput. Biol. Med..

[14] Elgendi M., Bobhate P., Jain S., Guo L., Rutledge J., Coe Y., Zemp R., Schuurmans S., Adatia I. (2014). Spectral analysis of the heart sounds in children with and without pulmonary artery hypertension. Int. J. Cardiol..

[15] Elgendi M., Bobhate P., Jain S., Guo L., Kumar S., Rutledge J., Coe Y., Zemp R., Schuurmans D., Adatia I. (2015). The unique heart sound signature of children with pulmonary artery hypertension. Pulm. Circ..

[16] Schnupp J., Nelken I., King A. (2011). Auditory Neuroscience: Making Sense of Sound.

[17] Rich S., Dantzker D.R., Ayres N.A. (1987). Primary pulmonary hypertension: A national prospective study. Annals Int. Med..

[18] Ivy D.D., Abman S.H., Barst R.J., Berger R.M.F., Bonnet D., Fleming T.R., Haworth S.G., Raj J.U., Rosenzweig E.B., Neick I.S. (2013). Pediatric pulmonary hypertension. J. Am. Coll. Cardiol..

[19] Hoeper M.M., Bogaard H.J., Condliffe R., Frantz R., Khanna D., Kurzyna M., Langleben D., Manes A., Satoh T., Torres F. (2013). Definitions and diagnosis of pulmonary hypertension. J. Am. Coll. Cardiol..

[20] Rich S. Executive summary from the World Symposium on Primary Pulmonary Hypertension 1998, Evian, France, September 6–10, 1998, cosponsored by the World Health Organization. http://www.nitricoxiderx.com/pulmonary-hypertension.

[21] Simonneau G., Robbins I.M., Beghetti M., Channick R.N., Delcroix M., Denton C.P., Elliott C.G., Gaine S.P., Gladwin M.T., Jing Z.-C. (2013). Updated clinical classification of pulmonary hypertension. J. Am. Coll. Cardiol..

[22] Remez R.E., Rubin P.E., Pisoni D.B., Carrell T.D. (1981). Speech perception without traditional speech cues. Science.

[23] Ellis D. Sinewave Speech Analysis/Synthesis in Matlab. http://www.ee.columbia.edu/ln/rosa/matlab/sws/.

[24] Elgendi M., Fletcher R.R., Norton I., Brearley M., Abbott D., Lovell N.H., Schuurmans D. (2015). Frequency analysis of photoplethysmogram and its derivatives. Comput. Methods Programs Biomed..

[25] Elgendi M. (2016). Optimal Signal Quality Index for Photoplethysmogram Signals. Bioengineering.

[26] Bonferroni C.E. (1936). Teoria statistica delle classi e calcolo delle probabilitá. Pubblicazioni del R Instituto Superiore di Scienze Economiche e Commerciali di Firenze.

[27] Holm S. (1979). A simple sequentially rejective multiple test procedure. Scand. J. Stat..

